# Single-Cell TCR Sequencing Uncovers Remodeling of the Immune Repertoire After a Short-Term Gluten-Free Diet in Pediatric Celiac Disease

**DOI:** 10.3390/ijms26188927

**Published:** 2025-09-13

**Authors:** Rafael Martín-Masot, Marta Herrador-López, Víctor Manuel Navas-López, Francisco David Carmona, Sara González-Muñoz, Elena López-Isac, Teresa Nestares, Lara Bossini-Castillo

**Affiliations:** 1 Sección de Gastroenterología y Nutrición Infantil, Hospital Regional Universitario de Málaga, 29010 Málaga, Spain; rafammgr@gmail.com (R.M.-M.); herradorlopezm@gmail.com (M.H.-L.); victor.navas@gmail.com (V.M.N.-L.); 2 Instituto de Nutrición y Tecnología de los Alimentos “José Mataix Verdú” (INYTA), Centro de Investigación Biomédica (CIBM), Universidad de Granada, 18071 Granada, Spain; nestares@ugr.es; 3 Departamento de Farmacología y Pediatría, Facultad de Medicina, Universidad de Málaga, 29071 Málaga, Spain; 4 Departamento de Genética e Instituto de Biotecnología, Centro de Investigación Biomédica (CIBM), Universidad de Granada, 18071 Granada, Spain; dcarmona@ugr.es (F.D.C.); saragonmu@ugr.es (S.G.-M.); 5 Reproducción Humana y Enfermedades Hereditarias y Complejas (IBS-TEC14), Terapias Avanzadas y Tecnologías Biomédicas, Instituto de Investigación Biosanitaria de Granada (ibs.GRANADA), 18012 Granada, Spain; 6 Departamento de Bioquímica y Biología Molecular II, Facultad de Farmacia, Instituto de Biotecnologia, Centro de Investigación Biomédica (CIBM), Universidad de Granada, 18071 Granada, Spain; lopezisac@ugr.es; 7 Departamento de Fisiología, Facultad de Farmacia, Universidad de Granada, 18071 Granada, Spain

**Keywords:** celiac disease, TCR, T cell, pediatric, gluten-free diet

## Abstract

Celiac disease (CD) is a chronic autoimmune disorder triggered by gluten in genetically susceptible individuals. While gluten-free diet (GFD) remains the primary treatment, the molecular mechanisms underlying immune reconstitution remain poorly understood in pediatric populations. This study aimed to characterize T cell receptor (TCR) repertoire remodeling in pediatric CD patients following short-term GFD. We conducted a longitudinal observational study analyzing peripheral blood circulating T cells from five pediatric CD patients at two time points: pre-GFD (at diagnosis) and post-GFD (after 9–10 months of strict dietary adherence). Single-cell TCR sequencing was performed to analyze clonotype diversity, gene usage patterns and TRAV-TRBV pairing combinations. Analysis of 9661 T cells revealed significant TCR repertoire remodeling post-GFD. Expanded clones, predominantly cytotoxic CD8+ T cells, contracted post-GFD (*p* = 0.02), while increasing clonotype diversity. Notably, specific αβ chain pairings underwent clear reorganization in the complete T cell compartment. Pathogenic combinations were depleted post-GFD, especially in CD4+ T cells, while beneficial pairings became enriched. GFD induced comprehensive TCR repertoire remodeling, revealing that changes occur at the level of specific TCR pairings rather than individual gene usage. Our findings highlight the precision of single-cell approaches in capturing functionally relevant immune changes for monitoring treatment response in pediatric CD.

## 1. Introduction

Celiac disease (CD) is a chronic autoimmune disorder triggered by the ingestion of gluten in genetically predisposed individuals, characterized by an immune-mediated enteropathy and a wide range of clinical manifestations [1]. Among cereals, wheat (*Triticum aestivum*) contains the highest concentration of immunoreactive gluten peptides responsible for triggering CD. Nevertheless, barley (*Hordeum vulgare*), rye (*Secale cereale*), and related hybrids are also significant sources. In pediatric populations, CD is particularly concerning due to its potential impact on growth, development, and long-term health outcomes [2]. The only currently available treatment is a strict, lifelong gluten-free diet (GFD), which has been shown to alleviate symptoms, promote mucosal healing and normalize serological markers in most patients [3]. However, the underlying immunological mechanisms driving the response to GFD, particularly in children, remain incompletely understood.

The immune response in CD is primarily mediated by adaptive immunity, with gluten-specific CD4+ T cells playing a central role in disease pathogenesis [4]. These T cells are active in multiple compartments relevant to disease, including T cells in the lamina propria, lymph nodes, and circulating T cells in peripheral blood. CD4+ T cells recognize gluten-derived peptides presented by HLA-DQ2 or HLA-DQ8 molecules, leading to the production of pro-inflammatory cytokines and subsequent tissue damage mediated mainly by activating cytotoxic CD8+ intraepithelial lymphocytes (IELs) [5]. The T cell receptor (TCR), which is responsible for antigen recognition, is a critical component of this process. The diversity of the TCR repertoire reflects the ability of the immune system to respond to a wide range of antigens, and alterations in TCR diversity have been implicated in various autoimmune and inflammatory conditions [6]. In CD, the TCR repertoire of gluten-specific T cells is thought to be shaped by both genetic and environmental factors, including gluten exposure [7]. While the intestinal mucosa harbors key effector T cell populations, our study focuses on circulating T cells obtained from peripheral blood, to characterize systemic immune changes after GFD intervention as the TCR repertoire evolution in response to GFD, particularly in pediatric patients, remains poorly characterized.

Recent advances in single-cell RNA sequencing (scRNA-seq) have revolutionized the study of immune cell heterogeneity and TCR repertoire diversity [8]. Unlike bulk sequencing approaches, scRNA-seq allows for the simultaneous analysis of gene expression and TCR clonality at single-cell resolution, providing unprecedented insights into the functional and clonal dynamics of T cell populations [9]. This technology has been successfully applied to study autoimmune diseases, including rheumatoid arthritis and type 1 diabetes, revealing novel insights into disease mechanisms and therapeutic targets [10,11]. In the context of CD, scRNA-seq offers a unique opportunity to explore the impact of GFD on the TCR repertoire and its relationship with clinical outcomes.

**Figure 1:**
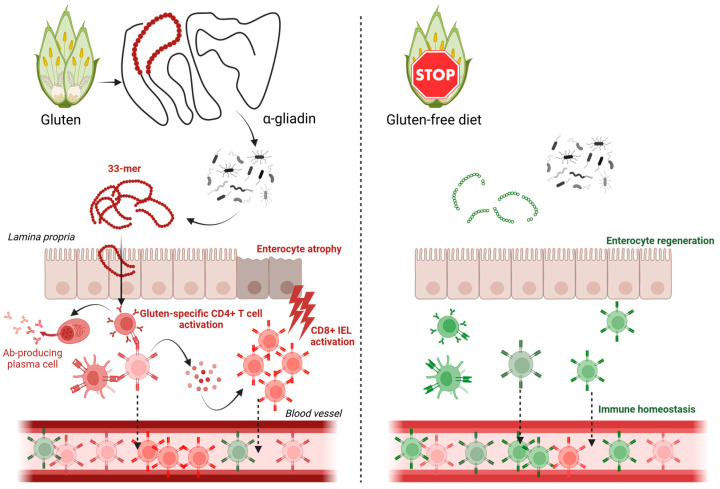
Gluten-Free Diet Modulates Immune Activation and Peripheral T Cell Repertoire in Celiac Disease. Partial gluten digestion in predisposed individuals triggers gliadin-derived peptide presentation, CD4+ T cell activation, cytokine release, and CD8+ intraepithelial lymphocyte (IEL) activation, leading to enterocyte atrophy and a shift in peripheral blood T cell receptor (TCR) repertoire. A gluten-free diet (GFD) halts these immune responses, allowing enterocyte regeneration and restoration of immune homeostasis in blood and tissue.

The GFD has been shown to modulate the immune response in CD, leading to a reduction in gluten-specific T cells and a restoration of mucosal homeostasis [12]. However, it is important to acknowledge that the TCR repertoire in circulating lymphocytes may differ significantly from the repertoire present in intestinal tissue, particularly among IELs, which are directly involved in disease pathology and often harbor expanded gluten-specific clones. Therefore, the extent to which GFD influences the diversity of the TCR repertoire in peripheral blood, particularly in pediatric patients, is not well understood. Some studies suggest that early initiation of GFD in children may lead to a more complete restoration of immune homeostasis compared to adults [13]. However, other studies have reported persistent immunological abnormalities even in patients adhering to a strict GFD, raising questions about the long-term efficacy of dietary therapy [14]. Understanding the impact of GFD on TCR diversity could provide valuable insights into the mechanisms underlying these observations and inform the development of more effective therapeutic strategies (Figure 1).

Despite the growing body of literature on CD and the GFD, significant gaps remain in our understanding of the immunological changes induced by dietary therapy, particularly in pediatric populations. Most studies have focused on serological and histological outcomes, with limited attention to the underlying T cell dynamics [15]. Furthermore, the use of scRNA-seq to study TCR diversity in CD is still in its infancy, with few studies exploring its potential in this context [16,17]. Addressing these gaps is critical for advancing our understanding of CD and improving patient care.

This study aims to investigate the effect of GFD on TCR repertoire diversity in the circulating peripheral blood T cells of pediatric CD patients using scRNA-seq, enabling non-invasive, longitudinal analysis in pediatric CD patients. By analyzing the TCR repertoire at single-cell resolution, we seek to characterize the changes in T cell clonality and diversity induced by GFD and to explore their relationship with clinical outcomes. Our findings will provide new insights into the immunological mechanisms underlying the response to GFD and may help identify biomarkers for predicting treatment response in pediatric CD patients.

## 2. Results

To investigate the impact of a GFD on the TCR repertoire in pediatric CD patients, we analyzed single-cell TCR sequencing data from five patients before and after 9–10 months of GFD. Below, we present the key findings, supported by detailed visualizations and statistical analyses, focusing on the comparison between pre-GFD and post-GFD groups.

### 2.1. Short-Term Gluten-Free Diet Increases the Frequency of Unique Clones in Cytotoxic CD8+ T Cells

**Figure 2:**
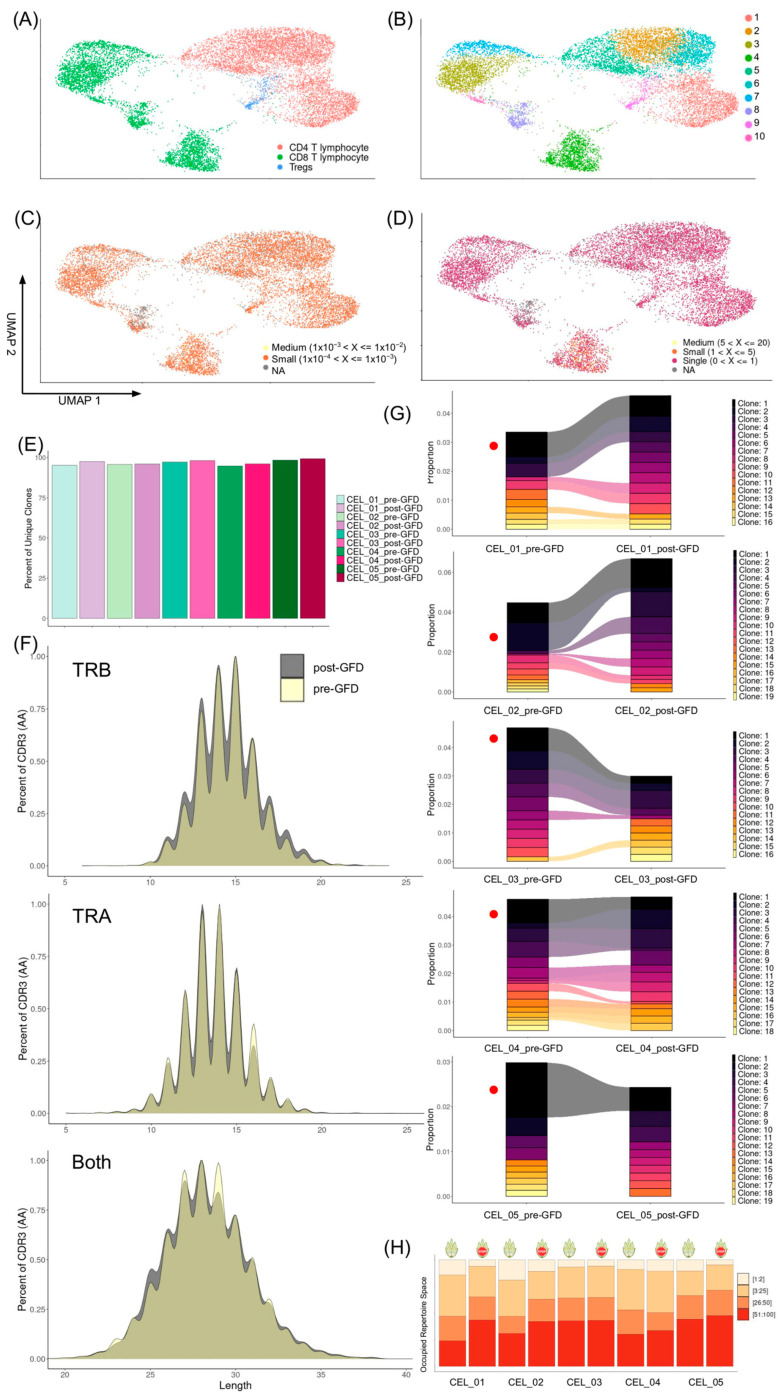
Clonal architecture, diversity, and persistence of T cells in peripheral blood from pediatric CD patients using single-cell transcriptomics, comparing pre-GFD and post-GFD states. Uniform Manifold Approximation and Projection (UMAP) plots of single-cell transcriptomes from pediatric CD patients, colored by (**A**) major T cell subsets, (**B**) high-definition sub-clusters, (**C**) clonal size proportions, (**D**) clonal size total numbers. (**E**) Barplots displaying the relative abundance of unique TCR clonotypes per individual stratified by dietary group (pre- or post-GFD). (**F**) Distribution of CDR3 amino acid lengths for both TRB, TRA and both chains across all samples, grouped by diet. (**G**) Alluvial diagrams for the top 10 clonotypes in paired samples per patient (CEL_01 to CEL_05), with flows tracking transitions; *x*-axis lists clones, *y*-axis shows pre- to post-GFD fate, showing persistence or loss (red dot: most frequent clone). (**H**) Barplots indicating the occupied repertoire space for each sample before and after GFD.

We analyzed the valid TCR sequences of 11,808 T cells that were classified as CD4+ (4 subclusters, 6902 cells), CD8+ (5 subclusters, 4545 cells) and regulatory T cells (1 subcluster, 361 cells) (Figure 2A,B and Figure S1, Table S1). Hyperexpanded clones (>100 cells) were not observed either pre-GFD or post-GFD. However, medium and small size clones were observed in a specific subcluster of CD8+ (Figure 2C,D), which corresponded to highly active cytotoxic T cells characterized by high expression of genes such as *CST7*, *CCL5*, *GZMA*, *NKG7*, and *GZMH* (Supplementary Table S2). Moreover, the presence of expanded clones was higher pre-GFD and, conversely, the percentage of unique cell clones increased in all patients post-GFD, showing an increased diversity (*p_t_*_-test_ = 0.02, Figure 2E, Table S3). Nevertheless, the relative abundance of clones did not show evident deviations pre- and post-GFD and the amino acid length of the CDR3 regions were not significantly different based on diet status (Figure 2F).

Interestingly, we observed that the most abundant clone in every patient pre-GFD was still present post-GFD, but it was clearly reduced. While we observed persistence and frequency shifts in several top 10 pre-GFD clones after the GFD, patient CEL_05, who was homozygous for HLA-DQ2.5, showed the most evident renewal of clones with new clones substituting the previous ones and a clear reduction in the most frequent clone after the GFD (Figure 2G and Figure S2). However, clonal proportions were not substantially altered by the GFD and only the frequency of the top clones seemed to be affected (Figure 2H). Additionally, we observed a homogeneous composition between individuals in the amino acid frequency per position both in the TRB and in the TRA chains, but we detected no significant changes before and after the GFD (Figure S2). Positional entropy was not affected by the GFD (Figure S3) and neither was the percentage of K-mers of nucleotides or amino acids in the CDR3 region (Figure S4).

**Figure 3:**
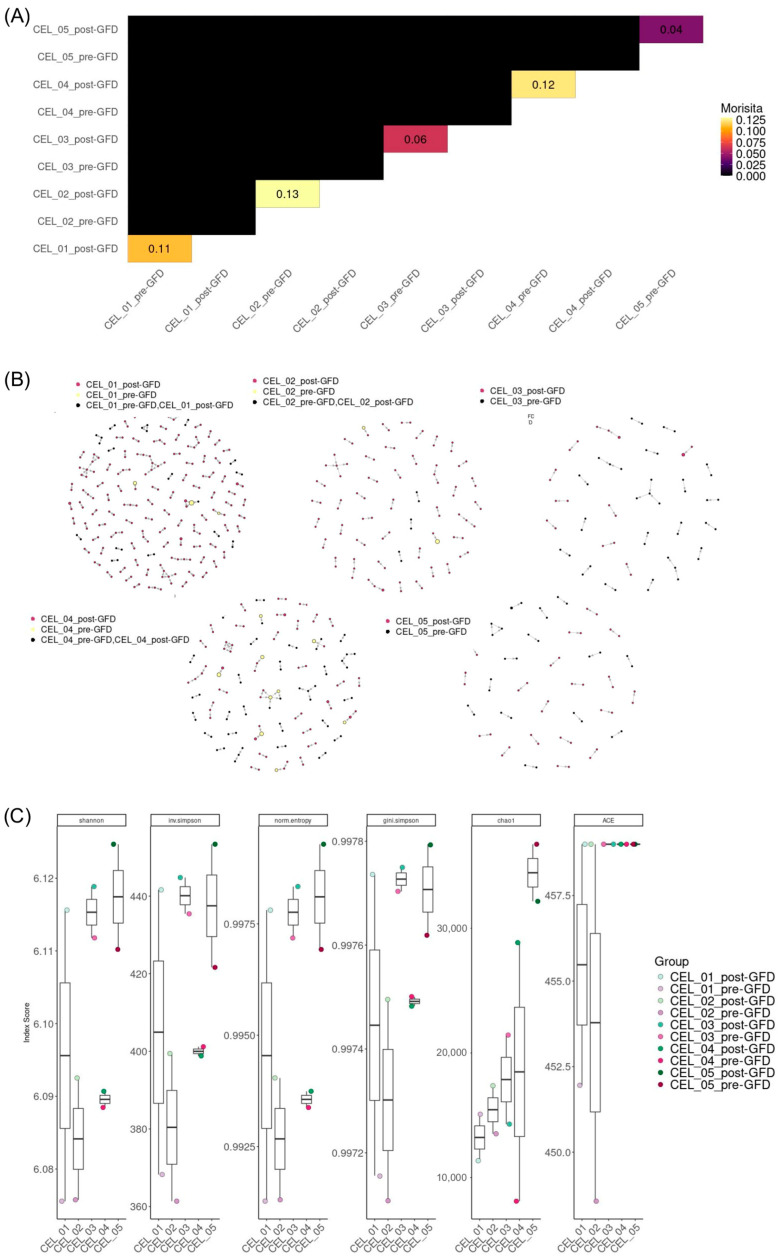
Repertoire overlap, V(D)J gene usage, and pairing architecture in pediatric CD pre- and post-GFD. (**A**) Heatmap of pairwise Morisita similarity indices, with color gradient showing TCR repertoire overlap. (**B**) Network representation, with nodes (circles labeled by sample and colored by sharing status) connected by edges (thickness proportional to shared clone count) of clones shared between conditions. (**C**) Box plots of diversity metrics (Shannon index, inverse Simpson, normalized entropy, Gini-Simpson, Chao1, and ACE) comparing pre- and post-GFD conditions; whiskers denote variability.

To assess the similarity between clonal repertoires across patients, we computed pairwise Morisita indices and visualized the results as a heatmap (Figure 3A). The Morisita index provides a quantitative measure of repertoire overlap, with values ranging from 0 (no overlap; entirely distinct repertoires) to 1 (complete overlap; identical repertoires including abundance distributions). The resulting matrix revealed that the majority of sample pairs exhibited minimal or no overlap, except for each patient pre- and post-GFD. This pattern demonstrates that most clonal repertoires were markedly distinct from one another, with few or no shared dominant clonotypes between individuals. Moreover, we observed only a partial overlap even in the same individual pre- and post-GFD.

In addition to monitoring the evolution of clones after the GFD, we were able to identify clonal clusters that were sharing amino acid sequences (Figure 3B). Although some clusters included both pre-GFD, post-GFD and shared clones, they showed different proportions and the majority of clusters were restricted to either pre-GFD or post-GFD.

### 2.2. TCR Repertoire Diversity Evenness Indices Increases Changes After a Gluten-Free Diet

We observed that metrics that capture both richness and evenness, i.e., Shannon entropy, inverse Simpson, normalized entropy, and Gini-Simpson, are consistently higher after treatment. This pattern reflects a repertoire where surviving clonotypes are more uniformly distributed, with diminished dominance of a few highly expanded clones. On the other hand, the Chao1 estimator, which emphasizes total unique clonotypes (including rare, unobserved ones), was reduced following treatment (Figure 3C, Table S4). This decrease suggested a contraction in the number of distinct TCR clonotypes, likely due to selective loss of rare clones or a repertoire bottleneck effect. These shifts in diversity parameters did not reach statistical significance (*p_t_*_-test_ > 0.05), but the tendency was maintained in all the individuals in the Shannon entropy and normalized entropy indices.

Considering only the cluster of cytotoxic CD8+ T cells, all clonal diversity indices, including Shannon entropy, inverse Simpson, normalized entropy, Gini-Simpson, and the Chao1 richness estimator, showed marked increases, indicating that this specific cluster shows an increase in unique clonotypes (Figure S5). Once again, the differences were not significant (*p_t_*_-test_ > 0.05), but the tendency was maintained in all the individuals except for CEL_04, who showed a more stable TCR repertoire pre- and post-GFD.

### 2.3. VDJ Gene Pairing Changes After a Gluten-Free Diet

Leveraging the capabilities of scRNA-seq, we were able to directly detect paired *TRAV* and *TRBV* gene usage, a level of resolution typically unattainable with bulk sequencing or less granular technologies. This advance allowed us to interrogate not only the individual gene usage frequencies but, uniquely, their specific pairings within single cells.

**Figure 4:**
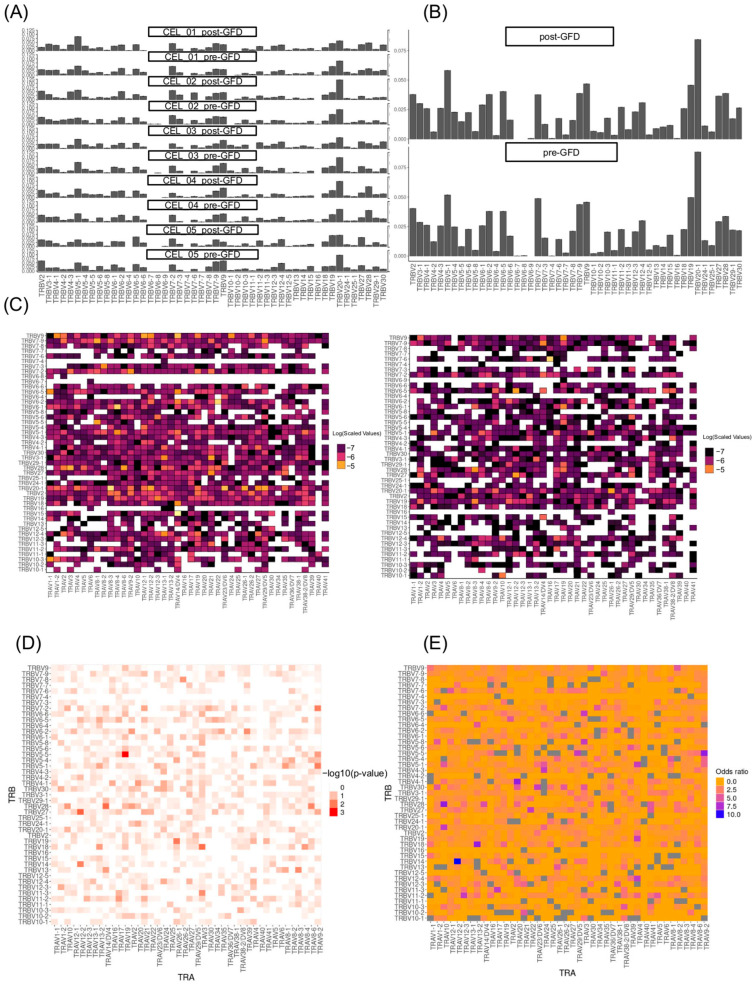
VJ-gene usage pre- and post-GFD. (**A**) Histogram of *TRBV* gene usage across individual pre- and post-GFD samples (bars for each gene with *y*-axis as proportion). (**B**) Aggregated histograms of *TRBV* gene usage pre- and post-GFD. (**C**) Heatmap of *TRAV* and *TRBV* gene pairing pre-GFD (left) and post-GFD (right), with rows/columns as genes and color intensity for frequencies. (**D**) Heatmap of *p*-values of the Fisher-test comparing the *TRAV–TRBV* pair frequency between dietary conditions. (**E**) Heatmap of odds ratios of the Fisher-test comparing the *TRAV–TRBV* pair frequency between dietary conditions.

Considering all T cells assayed, the overall distributions of individual *TRAV* and *TRBV* gene usage appear broadly similar between pre- and post-treatment conditions in all T cells, supporting our observation that marginal usage frequencies are largely stable (Figure 4A,B and Figure S6). However, the *TRAV-TRBV* pairings reveal a contrasting scenario, a marked reorganization in the frequency patterns of specific V gene combinations is evident when comparing the pre- and post-GFD repertoires. The heatmaps clearly visualize pairs such as *TRAV12-2-TRBV14* and *TRAV10-TRBV28*, identified as significantly enriched post-GFD (odds ratios > 8, *p*_Fisher_ < 0.05), as hotspots of increased pairing frequency. Conversely, combinations such as *TRAV2–TRBV6-2*, *TRAV26-2–TRBV7-2*, *TRAV2–TRBV7-2*, or *TRAV26-1–TRBV19*, depleted after intervention (odds ratios near zero), are equally evident as diminished or absent (Figure 4C–E, Table S5). Although the statistical significance of these changes is lost after multiple testing, the qualitative shift is further substantiated by the Cochran–Mantel–Haenszel test results, which showed a significant global difference in the pairing distribution (*p*_CMH_ < 2.2 × 10^−16^). Remarkably, the decrease in the frequency of pairs including the *TRBV7-2* gene was observed in CD4+ T, but not in CD8+ T cells, after subtype stratification (Table S5).

The pairings between *TRBV* and *TRBJ* or *TRAV* and *TRAJ* genes also showed pre- and post-GFD shifts that were confirmed by significant results in their corresponding Cochran–Mantel–Haenszel tests (Figure S6).

## 3. Discussion

The present study demonstrates alterations in the peripheral blood TCR repertoire architecture following short-term GFD intervention in CD patients, revealing complex patterns of immune remodeling that extend beyond conventional immunological metrics. Our findings collectively indicate a fundamental restructuring of the adaptive immune landscape, characterized by enhancement of repertoire evenness and a differential pairing of TRA and TRB genes.

The observed reduction in clonotype expansion among CD8+ T cells post-GFD represents a critical finding that aligns with established paradigms of CD pathogenesis. Functional annotation confirmed that the most prominently expanded clonotypes corresponded to cytotoxic CD8+ T cells, which might be consistent with the well-documented role of CD8+ IELS in gluten-mediated tissue damage [1]. The contraction of these expanded clones following dietary intervention likely reflects the cessation of chronic antigenic stimulation by gluten-derived peptides, particularly the immunodominant α-gliadin epitopes presented by HLA-DQ2/DQ8 molecules. Different studies have shown that there is a close relation between IELs and circulating CD8+ T cells in CD, with evidence showing that gluten exposure induces expansion of gluten-specific clones and their subsequent recruitment from circulation to the intestinal mucosa [18,19]. However, the present study provides the first comprehensive TCR repertoire analysis documenting the reconstitution dynamics following GFD intervention in pediatric CD patients. Although the complete extent of TCR repertoire shift in IELs may not be fully represented in the circulation, we showed that the dynamic trafficking between the gut and blood contribute to making peripheral blood not a perfect but a good proxy for TCR repertoire changes in the gut.

One of the most striking findings was the simultaneous increase in TCR repertoire diversity indices, including Shannon entropy and clonotype richness, alongside improved clonal evenness post-GFD. This apparent paradox, wherein clonal contraction coexists with enhanced diversity, reflects the complex dynamics of immune reconstitution. The increase in Chao1 diversity estimates suggests not merely the persistence of existing rare clones, but potentially the emergence or expansion of previously suppressed clonotypes. The enhanced clonal evenness, as evidenced by improved Simpson and Gini-Simpson indices, indicates a transition from an oligoclonal, gluten-focused response to a more balanced, surveillance-oriented repertoire. This shift toward greater repertoire evenness may represent optimal adaptive immune function, where no single clone dominates the landscape, thereby preserving the capacity for diverse antigenic responses. The increase in diversity and the reduction in clone sizes, also known as clonal contraction, parallels observations in other autoimmune conditions where treatment with autologous stem cells leads to the resolution of pathogenic T cell responses [20,21]. This emphasizes that immune reconstitution via renewal of TCR diversity is a fundamental feature of disease remission across multiple immune-mediated conditions. Thus, the immune repertoire changes we observe post-GFD in CD reflect a shared mechanism of immune resetting that transcends specific diseases. However, these results should be considered with caution since they corresponded to trends and did not reach statistical significance, probably due to the limited sample size.

Our findings converge with established knowledge in CD immunology while revealing previously hidden layers of TCR repertoire organization. The stability of individual *TRAV* and *TRBV* gene usage frequencies in our post-GFD analysis aligns with the broader patterns observed in longitudinal studies [22,23], where population-level gene usage remained relatively consistent despite significant changes in clonal diversity. This consistency suggests that the fundamental machinery of TCR generation and the basic selective pressures shaping V gene utilization are preserved even during profound immunological remodeling.

Our single-cell approach reveals an important limitation in previous bulk sequencing studies of CD. Traditional methods have identified certain TCR gene segments, particularly *TRBV7-2*, *TRBV6*, and *TRBV20*, as overused in gluten-reactive T cells [24]. However, when we examined how these genes are actually combined within individual cells, we discovered a more complex picture. While the overall usage of individual gene segments remained stable after GFD treatment, the specific ways these segments pair together changed dramatically. For example, the combination of different *TRAV26-TRBV7* genes in pairs, which previous studies identified as characteristic of gluten-reactive cells [24], was significantly reduced after dietary intervention. Interestingly, we observed that pairs carrying TRBV7-2, the TCR gene most associated with gluten, were reduced specifically in CD4+ T cells, highlighting their key role in the gluten-specific response. Additionally, the pairing *TRAV26-1-TRBV19* became depleted post-GFD in the complete T cell population. This finding is significant because it suggests that the pathogenic TCR repertoire in CD is defined not simply by the presence of certain gene segments, but by their specific combinations. Previous bulk sequencing approaches, which analyze gene usage in aggregate, may have missed this crucial layer of organization. Our results indicate that successful GFD treatment eliminates precisely those TCR combinations that earlier tetramer-based studies identified as gluten-specific, while preserving the overall capacity to use the same individual gene segments in different, potentially beneficial combinations.

The enrichment of novel pairings such as *TRAV12-2-TRBV14* in the complete T cell population, *TRAV12-2-TRBV27* especially in the CD4+, and *TRAV10-TRBV28* especially in the CD8+ T cells following GFD represents a fascinating counterpoint to the known gluten-reactive repertoire. These combinations, which were either rare or absent in the pre-GFD state, may represent the emergence of regulatory or homeostatic T cell populations that help maintain intestinal tolerance. This interpretation aligns with the documented increase in clonal diversity observed in longitudinal studies [22], but extends it by suggesting that diversity restoration involves not just the contraction of dominant clones but the active emergence of previously suppressed TCR specificities.

This mechanism, supported by the Morisita index analysis revealing largely unique clonal landscapes between pre- and post-GFD, explains how this extensive repertoire turnover achieves histological remission while retaining immune memory. The temporal dynamics from gluten challenge studies [22,23], where re-exposure rapidly expanded pre-existing memory populations with overlapping repertoires, gain new significance through our combinatorial lens.

Several limitations must be acknowledged in interpreting these findings. For example, the sample size, while adequate for single-cell analysis, may limit the generalizability of observed patterns across the broader CD population. The heterogeneity in HLA backgrounds and undetected heterogeneity among patients could influence repertoire dynamics in ways not fully captured by this analysis. While functional annotation identified expanded clonotypes as CD8+ T cells, the specific antigenic specificities of these clones remain unknown. Future studies incorporating peptide-MHC tetramer staining or TCR reconstruction approaches would provide direct evidence for gluten-specific responses. Moreover, likely due to limited cell numbers, we were not able to detect TCR repertoire changes in scarce T cell subsets such as regulatory T cells.

In conclusion, this study demonstrates that GFD intervention in CD induces comprehensive TCR repertoire remodeling characterized by the contraction of pathogenic CD8+ T cell clones and the restoration of balanced immune diversity. The correlation between increased clonotype diversity post-GFD and improved clinical outcomes represents a significant advance in understanding the relationship between immune repertoire architecture and disease resolution. These findings suggest that TCR repertoire metrics, including diversity and clonality, could serve as sensitive biomarkers to complement conventional antibody titers and clinical symptom evaluation, thereby enhancing patient monitoring. Incorporating TCR repertoire profiling into clinical practice may enable personalized follow-up and therapeutic optimization, aligning with emerging trends in precision medicine for autoimmune disorders.

## 4. Materials and Methods

### 4.1. Study Design and Patient Description

We conducted a longitudinal observational study involving five pediatric patients diagnosed with CD. The cohort included four females and one male, aged between 8 and 14 years at diagnosis, all of European descent (see Table 1 for detailed demographic and clinical characteristics). Patients met the diagnostic criteria established by the European Society for Pediatric Gastroenterology Hepatology and Nutrition (ESPGHAN) 2020 guidelines [2]. Briefly, diagnosis was based on serology (positive anti-TTG2 IgA or anti-endomysial antibodies), compatible HLA-DQ2/DQ8 carrier status, and histology of duodenal villous atrophy, with biopsy sometimes omitted in strongly seropositive, non-symptomatic cases. Anti-TTG2 IgA antibodies were measured by ELISA (Phadia™ Thermo Fisher Scientific, Waltham, MA, USA), with the cutoff point for a positive result in the anti-TTG2 IgA assay being 7 U/mL, in accordance with the manufacturer’s instructions. Key exclusion criteria comprised acute infections at sampling, chronic hepatic, pulmonary, rheumatologic, or renal diseases, obesity (as per International Task Force criteria), inflammatory bowel disease, diabetes, or refusal to consent [25].

Patients were enrolled from the Gastroenterology and Pediatric Nutrition Unit of the Hospital Regional Universitario Málaga. Written informed consent was obtained from legal guardians, and all samples were irreversibly anonymized in compliance with EU Directive 2001/20/EC and national data protection regulations. Adherence to a gluten-containing diet (pre-GFD) or to a GFD (post-GFD) was confirmed by clinical evaluation and relevant testing, as described below.

Blood samples were collected at two time points: at diagnosis while patients were still consuming gluten (pre-GFD), and after 9 to 10 months of strict GFD adherence (post-GFD). To objectively assess dietary compliance, fecal samples were collected at two occasions, once during a working day and once during a non-working day at approximately four months and shortly before blood sampling. These were analyzed for gluten immunogenic peptides (GIP) using the iVYCHECK GIP Stool kit (Biomedal S.L., Seville, Spain, Catalog Ref: KT-5737), which has demonstrated a sensitivity of 95–100% and specificity of 100 [26,27], to confirm absence of gluten intake. Additionally, a detailed 24 h dietary recall was performed over three non-consecutive days, including at least one non-working day, under the supervision of a trained dietitian. Samples were categorized into two groups: pre-GFD (patients consuming gluten with positive AATG-IgA) and post-GFD (patients on a strict GFD with negative GIP tests) [28]. All patients showed minor intestinal symptoms at debut.

### 4.2. PBMC Isolation and Cryopreservation

Thirty milliliters of peripheral blood were collected from each participant in EDTA tubes (Greiner Bio-one #4550356, Kremsmünster, Austria) and processed within 3 h of collection. PBMCs were isolated using Ficoll^®^ Paque Plus density gradient centrifugation (Merck #GE 17-1440-02, Rahway, NJ, USA) following the manufacturer’s protocol. Cell suspensions were cryopreserved in 10% dimethyl sulfoxide (DMSO, Merck D2438) and 90% fetal bovine serum (FBS, Gibco #10082-147, Brisbane, Australia) and stored at −80 °C for at least 24 h before being transferred to liquid nitrogen for long-term preservation.

### 4.3. Single-Cell TCR Sequencing and Library Preparation

For TCR sequencing, cryopreserved PBMCs were thawed, washed, and manually counted using a Neubauer chamber (Thermo Fisher Scientific, Waltham, MA, USA). Viable cells were processed using the Chromium Next GEM Single Cell 5′ Library and Gel Bead Kit v2 (PN-1000265, 10x Genomics, Pleasanton, CA, USA) and the Chromium Next GEM Chip G Single Cell Kit (PN-1000127, 10x Genomics, Pleasanton, CA, USA) following the manufacturer’s instructions. While the core emphasis of this article is on the TCR repertoire, this dataset is paired with transcriptomic profiling of the same cells. Therefore, we leveraged the full transcriptome data to classify T cells into distinct subsets (e.g., CD4+, CD8+, regulatory T cells), which informed our analyses of clonal dynamics and phenotypes as described in Martín-Masot et al. [29]. The human TCR-specific kit from 10x Genomics was used to capture TCR α and β chain sequences. Cell pools were prepared from randomly distributed samples to control for batch effects. TCR libraries were sequenced on a NovaSeq 6000 platform (Illumina, San Diego, CA, USA), generating an average of >80% reads in TCR genes, >5900 reads per cell. The VDJ gene reference corresponding to the GRCh38 genome build was used as the alignment reference, and unique molecular identifier (UMI) counts were generated using the Cell Ranger Single Cell Software Suite (v3.0.0, 10x Genomics) with default parameters.

### 4.4. TCR Repertoire Analysis Using scRepertoire

TCR sequences were demultiplexed and assigned to individual cells using the Cell Ranger output. Cells with fewer than 200 genes or more than 3500 mitochondrial genes in previous whole transcriptome analyses were excluded. Doublets were removed using the vireo package and genome-wide genotype data per donor [30].

The analytical workflow adhered to the established scRepertoire pipeline for single-cell immune receptor profiling [31], seamlessly integrated with Seurat v5 [32] as benchmark tool for transcriptomic analysis, employing default parameters with minimal adaptations such as chain filtering to ensure data fidelity. The pipeline included the following steps:

Clonotypes were defined based on unique combinations of TCR α and β chain sequences. Cell barcodes from TCR data were standardized and merged with scRNA-seq metadata, retaining only barcodes with available donor time point information. Barcodes containing more than two chains were filtered, keeping the top two most expressed chains per cell. The scRepertoire function *combineTCR()* was used to merge TCR sequences from different samples, ensuring consistent clonotype annotation across the dataset. These contig lists were then combined across samples with the *combineTCR()* function to create a unified object representing the full TCR repertoire across conditions and time points. Parameters included removal of missing data (*removeNA = TRUE*), removal of barcodes with more than two chains (*removeMulti = TRUE*), and selection of the two chains with highest expression (*filterMulti = TRUE*).

Clonotype information was integrated with single-cell transcriptomic data by linking TCR barcodes to the filtered subset of T cells preselected from previous analysis of the matched whole transcriptome dataset using the *combineExpression()* function. Clonotypes were defined based on gene calling (“gene”), grouped by sample, and clonal proportions were computed.

Quantitative measures of clonal diversity were derived using the following functions from scRepertoire: *clonalQuant()* quantified the number of unique clonotypes per sample and by experimental condition (diet); *clonalAbundance()* characterized the relative abundance and distribution of clones; *clonalLength()* assessed the CDR3 length distributions for TCR α and β chains; *clonalCompare()* performed pairwise comparisons of top clones (top 10) between pre- and post-GFD samples visualized with alluvial plots; *clonalScatter()* visualized the correlation of clone proportions between paired samples.

Diversity metrics including Shannon, Simpson, normalized entropy, Gini-Simpson, Chao1, and ACE indices were calculated using the *clonalDiversity()* function, with bootstrapping and downsampling to account for differences in repertoire sizes. Differences between dietary conditions were evaluated using paired *t*-tests on diversity estimates.

Repertoire size distributions were examined using *clonalSizeDistribution()*. The latter applied a spliced discrete gamma-GPD threshold model to characterize clone size distributions.

Repertoire overlap between samples was assessed using *clonalOverlap()* applying multiple similarity indices such as Morisita.

To characterize the molecular features of clonotypes, *percentAA()* quantified amino acid usage frequencies along the CDR3 sequences, *positionalEntropy()* calculated residue-wise sequence diversity and entropy, and *positionalProperty()* assessed biochemical properties of amino acids along CDR3 regions using Atchley factors.

Gene segment usage frequencies (V and J genes) were quantified via *percentGenes()*. Visualizations of gene usage distributions were generated with *vizGenes()*, producing heatmaps and barplots stratified by diet groups. T cell receptor alpha (TRAV) and beta (TRBV) gene pairing incidences were summarized in two-dimensional contingency tables, stratified by experimental condition (pre- and post-GFD). Global differences in TRAV-TRBV pairings between conditions were evaluated using the Cochran–Mantel–Haenszel test on three-dimensional contingency arrays, with statistical significance set at *p* < 0.05. For each TRAV-TRBV combination, local differences were further explored by constructing 2 × 2 tables of presence and absence across conditions, to which Fisher’s exact test and odds ratio calculations were applied. Adjusted *p*-values were computed to account for multiple comparisons. Data preprocessing and statistical analyses were carried out in R, with visualization of gene usage and pairing frequencies accomplished using the ggplot2 package.

Motifs within nucleotide and amino acid CDR3 sequences were characterized using *percentKmer()*, enumerating trinucleotide and tri-amino acid substrings to detect recurrent sequence features.

Clonotypes were clustered based on normalized Levenshtein edit distance of TRA amino acid sequences with *clonalCluster()*, applying a similarity threshold of 0.85. Clustering was performed per patient and sample group to identify networks of closely related clones. The resulting networks were visualized using igraph plotting functions with node sizing proportional to clone size and coloring by sample group.

## Figures and Tables

**Table 1:** Patient characteristics and TCR cell counts after QC.

	CEL_01	CEL_02	CEL_03	CEL_04	CEL_05	Total
T cells with TCR data	3489	2062	2150	2534	1573	11,808
Sex	F	M	F	F	F	
Age at diagnosis	10	12	13	14	8	
HLA-DQ2.5 copies	1	1	1	1	2	
Marsh	IIIB	IIIB	IIIA	IIIB	-	
Antiendomysial (qualitative)	Positive	Positive	-	Positive	Positive	
Antitransglutaminase baseline (U/mL)	9.9	74	35	87	255	
Antitransglutaminase follow-up (U/mL)	1.32	5.66	15	13	2.24	
BMI	13.45	18.66	20.08	25.32	15.68	
GIP 4 months	Negative	Negative	Negative	Negative	Negative	
GIP 9–10 months	Negative	Negative	Negative	Negative	Negative	

## Data Availability

The raw and processed TCR sequencing data, along with the R (version 4.4.2) scripts used for analysis, will be made available upon request pending approval from the corresponding author.

## Supplementary Materials

The following supporting information can be downloaded at https://www.mdpi.com/article/10.3390/ijms26188927/s1.

## Abbreviations

The following abbreviations are used in this manuscript:

CD	Celiac disease
GFD	Gluten-free diet
TCR	T cell receptor
GIP	Gluten immunogenic peptide
