# Supplementary figures and images for: Single-Cell TCR Sequencing Uncovers Remodeling of the Immune Repertoire After a Short-Term Gluten-Free Diet in Pediatric Celiac Disease
Source: Int J Mol Sci. 2025 Sep 13;26(18):8927. doi: 10.3390/ijms26188927 (PMC12469983; doi:10.3390/ijms26188927)

Figure S1

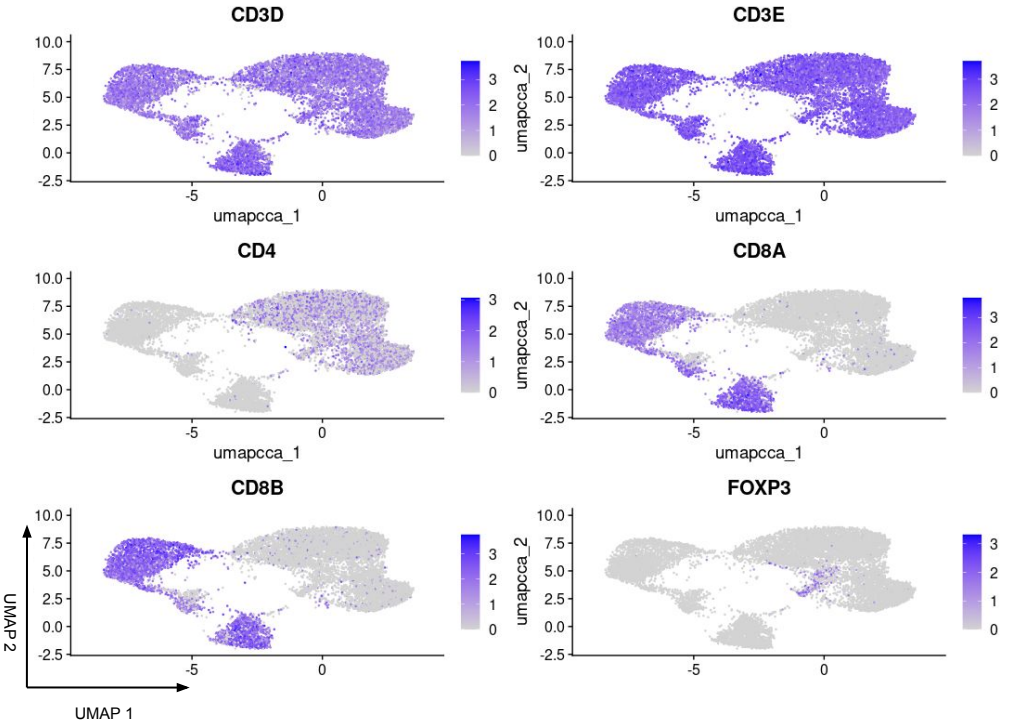

Figure S2

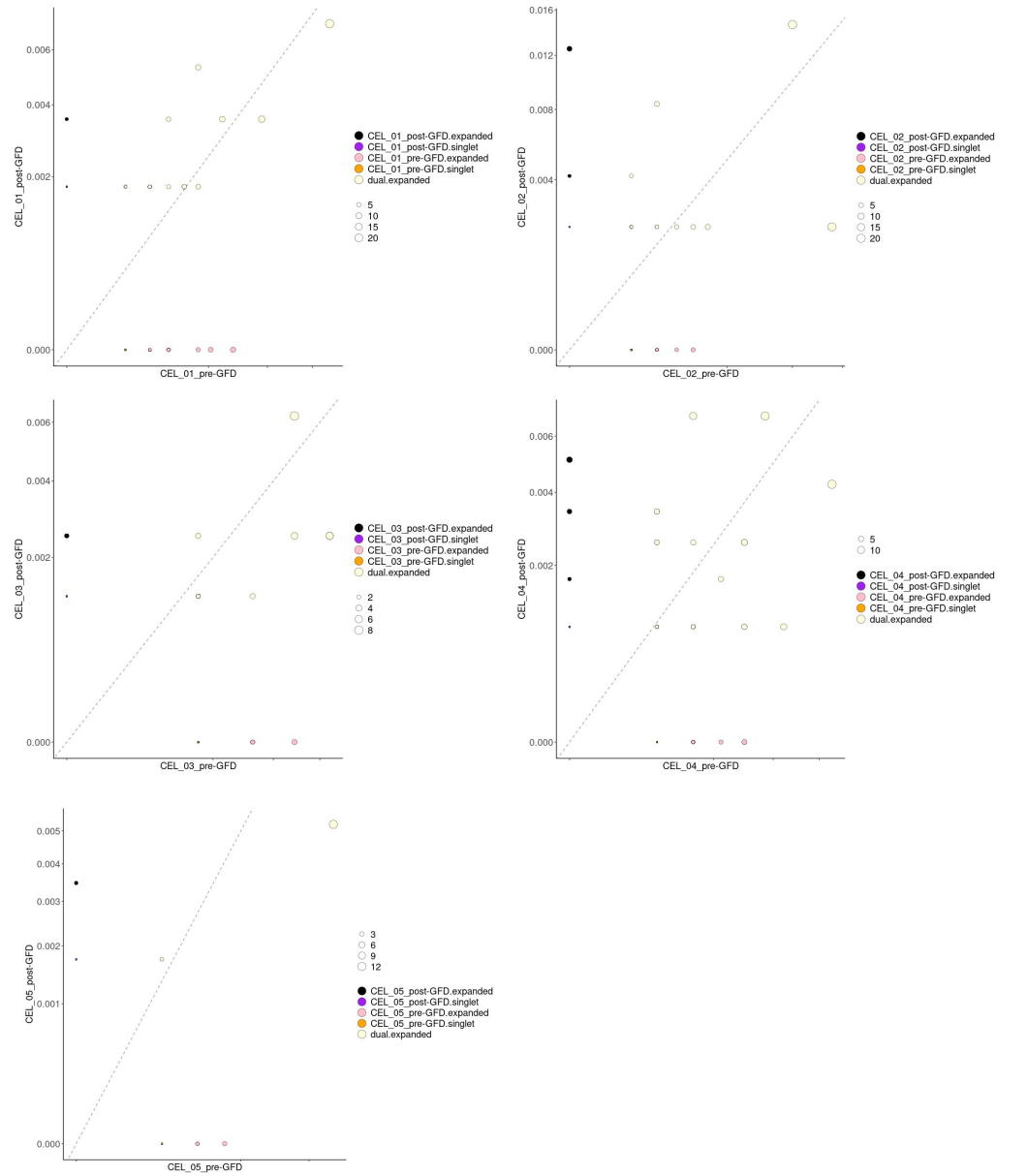

Figure S3

A)

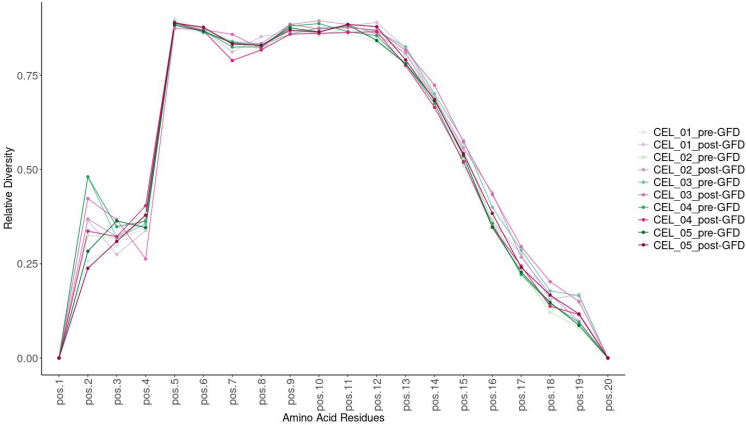

B)

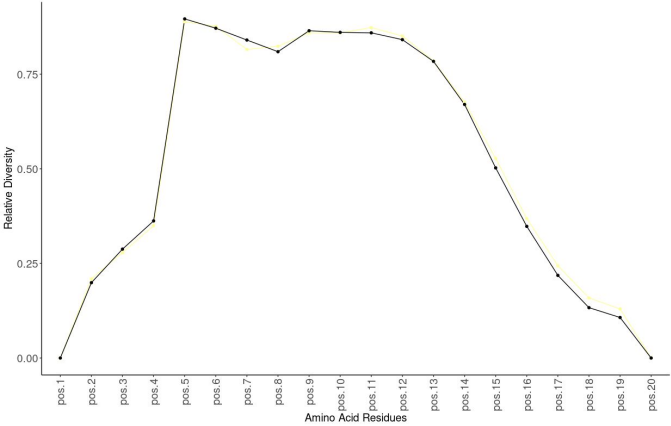

C)

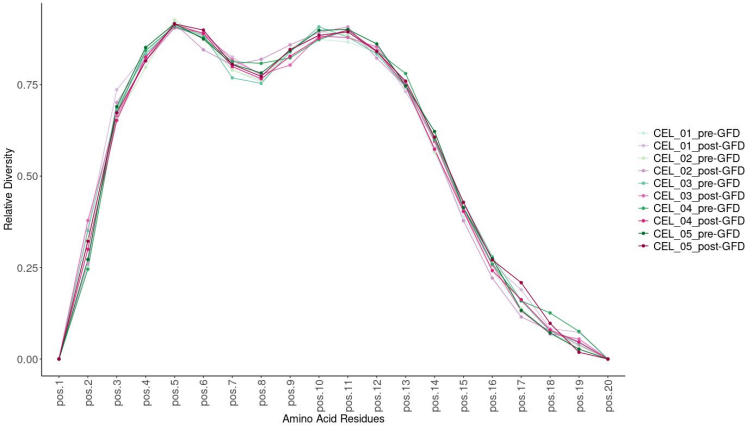

D)

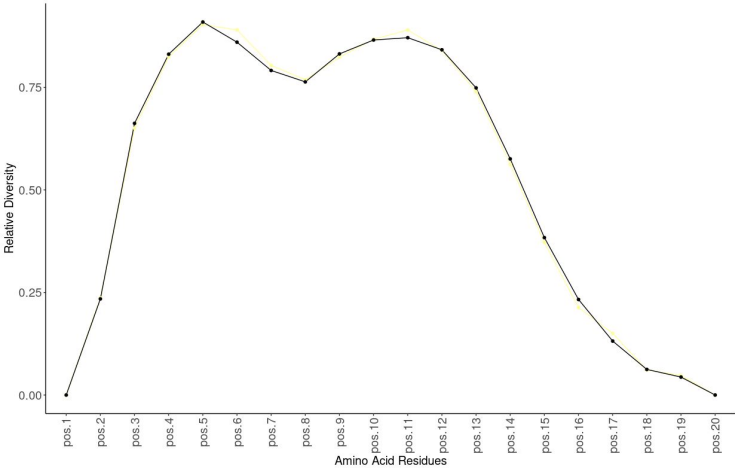

Figure S4

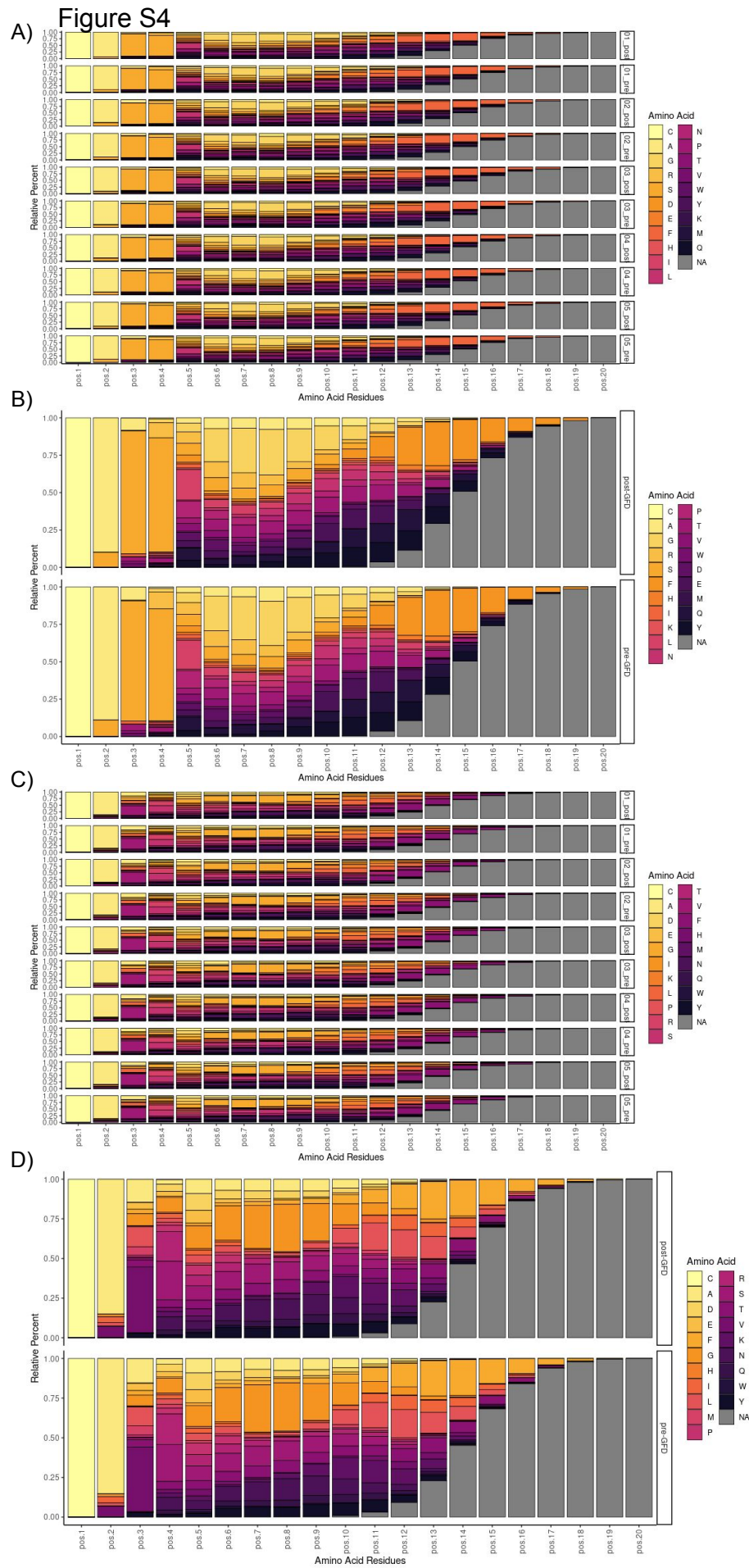

Figure S5

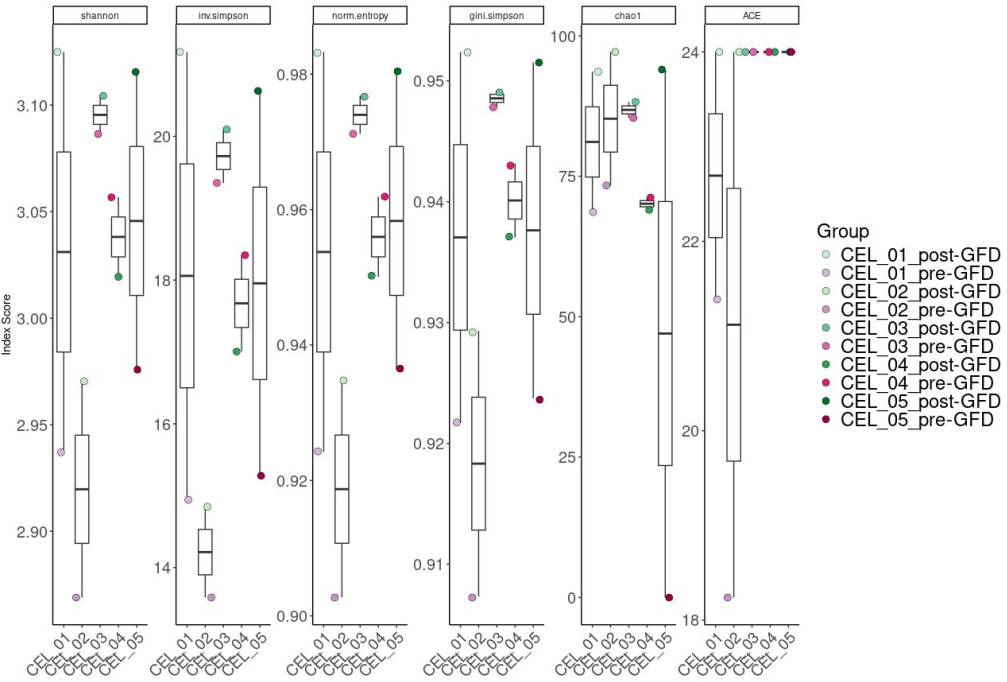

A) Figure S6

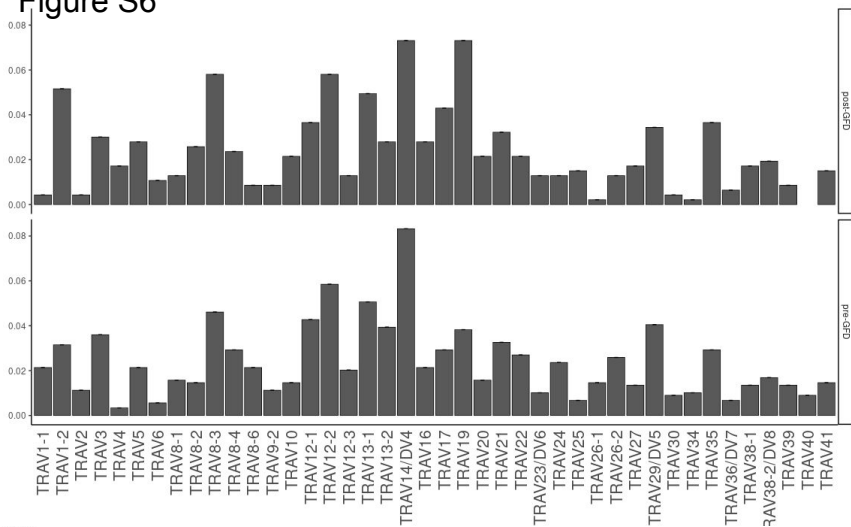

B)

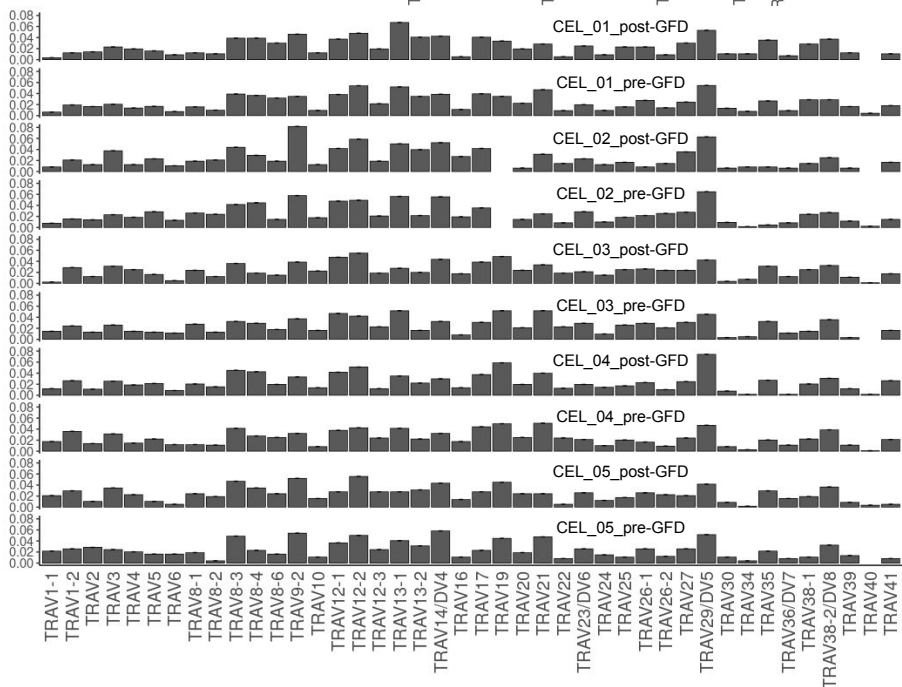

C)

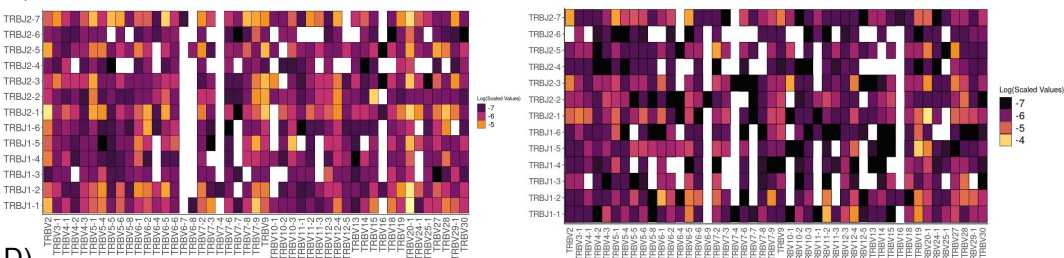

D)

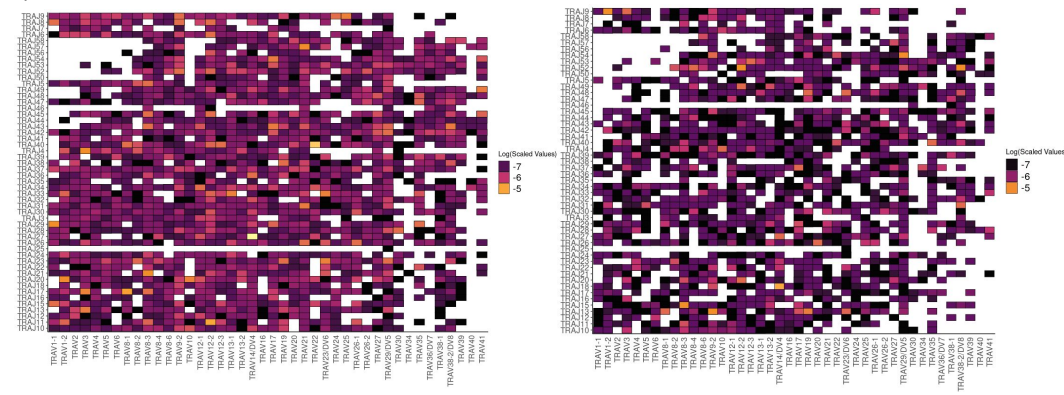

Supplement: Supplementary file 1 [file ijms-26-08927-s001.zip › Supplementary_Figures.pdf]
